# Pain referral patterns of the C1-C3 nerve roots: implications for headache disorders and the development of new therapies

**DOI:** 10.1186/1129-2377-14-S1-P49

**Published:** 2013-02-21

**Authors:** M Johnston, S Jordan, A Charles

**Affiliations:** 1 UCLA School of Medicine, USA

## Introduction

The upper cervical nerve roots (C1-3) are increasingly viewed as an important target for therapeutic intervention in headache, but their specific roles in the pathophysiology of head pain remain uncertain. An increased understanding of the role of C1-3 in primary and secondary headache disorders is important for progress with diagnostic and therapeutic interventions involving these structures.

## Objectives

The Objectives of this study are to characterize the distribution of pain provoked by stimulation at the C1-3 levels, and to investigate the potential efficacy of a novel, brief low temperature radiofrequency rhizolysis (BLT-RF) as a therapy for patients with occipital neuralgia.

## Methods

This study is a retrospective review of data from 9 patients with occipital neuralgia (5 of whom also had migraine) who underwent fluoroscopically guided multi-modal provocation at the C1, C2, and C3 levels followed by nerve root block with anesthetic and steroid. 7 patients underwent subsequent BLT-RF of the C1 spinal nerve and C2 and C3 dorsal root ganglia.

## Results

Patients with migraine all reported retro-orbital or periorbital pain with C1 stimulation. By contrast, patients without migraine reported occipital or cervical pain with stimulation at the C1 level. C2 and C3 stimulation evoked pain in occipital and cervical distributions similar to those previously reported. BLT-RF produced sustained pain relief in patients who had only transient relief with nerve block.

## Conclusions

The orbital/peri-orbital pain evoked by stimulation at the C1 level indicates that the C1 nerve root may play an important role in conditions in which pain occurs in this distribution, including migraine and cluster headache. The C1 nerve root may therefore be an important target for therapy for these conditions. The BLT-RF technique appears to be a safe and effective therapeutic approach to occipital neuralgia, with a longer duration of action than nerve blockade with anesthetics and steroids.
